# Analysis of Potential Vitamin D Molecule Biomarkers: Association of Calcitriol and Its Hydroxylation Efficiency Ratio with Cardiovascular Disease Risk in Rheumatoid Arthritis Patients

**DOI:** 10.3390/biomedicines12020273

**Published:** 2024-01-25

**Authors:** Melissa Rivera-Escoto, Bertha Campos-López, Karen Pesqueda-Cendejas, Adolfo I. Ruiz-Ballesteros, Paulina E. Mora-García, Mónica R. Meza-Meza, Isela Parra-Rojas, Edith Oregon-Romero, Sergio Cerpa-Cruz, Ulises De la Cruz-Mosso

**Affiliations:** 1 Red de Inmunonutrición y Genómica Nutricional en las Enfermedades Autoinmunes, Departamento de Neurociencias, Centro Universitario de Ciencias de la Salud, Universidad de Guadalajara, Guadalajara 44340, Jalisco, Mexico; melissa.rivera.e@hotmail.com (M.R.-E.); bertha.campos@live.com (B.C.-L.); karen.pesqueda20@gmail.com (K.P.-C.); adolfo.ruba@gmail.com (A.I.R.-B.); moragarciapaulinaesmeralda@gmail.com (P.E.M.-G.); monimez28@hotmail.com (M.R.M.-M.); iprojas@yahoo.com (I.P.-R.); 2 Instituto de Neurociencias Traslacionales, Departamento de Neurociencias, Centro Universitario de Ciencias de la Salud, Universidad de Guadalajara, Guadalajara 44340, Jalisco, Mexico; 3 Laboratorio de Investigación en Obesidad y Diabetes, Facultad de Ciencias Químico-Biológicas, Universidad Autónoma de Guerrero, Chilpancingo de los Bravo 39087, Guerrero, Mexico; 4 Instituto de Investigación en Ciencias Biomédicas, Centro Universitario de Ciencias de la Salud, Universidad de Guadalajara, Guadalajara 44340, Jalisco, Mexico; oregon_edith@hotmail.com; 5 Departamento de Reumatología, O.P.D. Hospital Civil de Guadalajara Fray Antonio Alcalde, Guadalajara 44280, Jalisco, Mexico; sacer04@prodigy.net.mx

**Keywords:** calcidiol, calcitriol, soluble vitamin D receptor, CVD risk, C-reactive protein, rheumatoid arthritis

## Abstract

Rheumatoid arthritis (RA) is a multifactorial autoimmune disease in which hypovitaminosis D by calcidiol quantification has been associated with disease severity. However, other vitamin D molecules could be implicated in RA pathophysiology and its comorbidities such as cardiovascular disease (CVD), which impacts the severity and mortality of RA patients. This study aimed to assess the relationship between calcidiol, calcitriol, its hydroxylation efficiency ratio, and the soluble vitamin D receptor (sVDR) and clinical and CVD risk variables to propose potential vitamin D molecule biomarkers for RA. A cross-sectional study of females was conducted on 154 RA patients and 201 healthy subjects (HS). Calcidiol, calcitriol, and the sVDR were measured in blood serum, and vitamin D hydroxylation efficiency was estimated using the calcitriol/calcidiol ratio score. CVD risk was calculated by the high-sensitivity C-reactive protein (hs-CRP) cutoff values. Disease activity was evaluated with the Disease Activity Score for 28 standard joints (DAS28-CRP). Results: The hydroxylation efficiency ratio and calcitriol serum levels were higher in RA patients with hypovitaminosis D (*p* < 0.001). Moreover, RA patients had a higher probability of a high hydroxylation efficiency ratio (OR = 2.02; *p* = 0.02), calcitriol serum levels (OR = 2.95; *p* < 0.001), and sVDR serum levels (OR = 5.57; *p* < 0.001) than HS. This same pattern was also observed in RA patients with high CVD risk using CRP serum levels; they showed a higher hydroxylation efficiency ratio (OR = 4.51; *p* = 0.04) and higher calcitriol levels (OR = 5.6; *p* < 0.01). Calcitriol correlates positively with the sVDR (r = 0.21, *p =* 0.03), CRP (r = 0.28, *p* < 0.001), and cardiometabolic indexes (*p* < 0.001) also showed discrimination capacity for CVD risk in RA patients with CRP ≥ 3 mg/L (AUC = 0.72, *p* < 0.01). In conclusion, hypovitaminosis D in RA patients was characterized by a pattern of a higher hydroxylation efficiency ratio and higher calcitriol and sVDR serum levels. Notably, higher calcitriol serum levels and a higher vitamin D hydroxylation efficiency ratio were associated with higher CVD risk in RA patients.

## 1. Introduction

Rheumatoid arthritis (RA) is one of the most prevalent chronic autoimmune diseases. Its pathogenesis involves mainly joint damage; however, other chronic systemic comorbidities such as cardiovascular disease (CVD) impact the severity and mortality of RA patients [1]. Several factors contribute to the onset, progression, and severity of disease; previous studies have suggested the potential role of vitamin D and molecules associated with its metabolism in the immunomodulation of inflammatory events.

It is worth noting that vitamin D is not a single compound but refers to a group of several molecules, which are derived from cholesterol via complex pathways of enzymatic and non-enzymatic reactions that participate in its metabolism [2]. Circulating cholecalciferol and ergocalciferol are activated by hydroxylation reactions; in the liver, the cytochrome P450 CYP2R1 hydroxylates these molecules to produce calcidiol, the most abundant inactive vitamin D molecule in the blood. Subsequently, in the kidneys, the cytochrome P450 CYP27B1 from calcidiol synthesizes the active vitamin D molecule; calcitriol, which exerts its genomic and non-genomic effects through vitamin D receptor (VDR) signaling [3]. According to a meta-analysis, hypovitaminosis D is associated with high RA disease activity [4]. Moreover, the evidence suggests that vitamin D could exert immunomodulatory and cardioprotective functions [5]. Notably, systematic reviews have reported the association between hypovitaminosis D and increased CVD risk, supported by an inverse association between calcidiol serum levels and cardiovascular events and mortality [6,7,8].

Although C-reactive protein (CRP) plays an important role in host defense mechanisms against infectious agents and the inflammatory response, the Centers for Disease Control and Prevention and the American Heart Association have proposed the high-sensitivity (hs-CRP) assessment as an inflammatory biomarker for consideration as a predictor of CVD risk [9]. In RA patients, hs-CRP is routinely assessed as a biomarker of unspecific systemic inflammation, and its high serum levels play a negative role in RA pathophysiology and proatherogenic events [10]. In other studies, it has been proposed that sufficient vitamin D serum levels might reduce CVD risk [7], perhaps due to the decrease in CRP serum levels. Furthermore, a negative correlation has been described between calcidiol and CRP serum levels [11]. Although we cannot establish the causal relationship of this association because the exact biological mechanisms are unknown, several studies support the positive role of calcidiol sufficiency in a healthy cardiometabolic profile [12].

Previously, the calcitriol/calcidiol ratio assessment has been reported in systemic lupus erythematosus (SLE) patients with hypovitaminosis D, which represents how many picograms of calcitriol are produced per nanogram of serum calcidiol as a representative score of the vitamin D hydroxylation efficiency ratio. Moreover, high calcitriol serum levels were related to pro-inflammatory variables in these autoimmune patients [13]. Additionally, some studies have reported the presence of the soluble VDR (sVDR) in different chronic and autoimmune conditions, but the mechanisms of why this nuclear receptor is in serum remain unknown to date [14,15,16,17,18] The presence of the sVDR in serum could be an indicator of a high rate of cell damage or apoptosis, characteristic of autoimmune diseases. However, more studies are required to elucidate this hypothesis [19].

According to this background, changes in the concentration of calcidiol, calcitriol, its hydroxylation efficiency ratio, and the sVDR could have potential pro-inflammatory and cardiometabolic roles in pathologic conditions; however, these molecules have not been evaluated together in RA patients. Therefore, this study aimed to assess the relationship between calcidiol, calcitriol, its hydroxylation efficiency ratio, and the sVDR and clinical and CVD risk variables to propose potential vitamin D molecule biomarkers for RA.

## 2. Materials and Methods

This cross-sectional study included a total of 154 female RA patients and 201 female healthy subjects (HS). The RA patients recruited were individuals who regularly attended the rheumatology service of Antiguo Hospital Civil de Guadalajara “Fray Antonio Alcalde”, during the period from January 2020 to August 2023. RA patients were previously diagnosed according to the 2010 classification criteria by the American College of Rheumatology/European League Against Rheumatism (ACR/EULAR 2010) [20]. In the case of HS, they were enrolled through invitations extended to the general population. All the participants reported no recent infections, surgeries, pregnancy, or autoimmune diseases in themselves or their relatives.

### 2.1. Ethical Considerations

Before the enrollment, each participant signed an informed consent form. The study protocol was previously approved by the Research Ethical Committee of the Hospital Civil Fray Antonio Alcalde (CEI. 135/23). Likewise, the entire study was carried out considering the international research guidelines.

### 2.2. RA Clinical Disease Activity Indexes

The Disease Activity Score for 28 standard joints (DAS-28) was used to evaluate the disease activity in RA, in addition to its derivations DAS28-CRP and erythrocyte sedimentation rate (ESR). A score of DAS-28 < 2.6 indicates remission and a score of DAS-28 ≥ 2.6 indicates disease activity in RA patients [21].

### 2.3. Quantification of Calcidiol, Calcitriol, sVDR, CRP, and Biochemical Variables

The biomolecules were measured in serum. Blood samples were obtained from each patient from an antecubital venipuncture, collected in the morning between 7:30 and 10:00 a.m. after an overnight fast (12 h), and then centrifuged for 10 min to obtain serum. Calcidiol, calcitriol, and the sVDR were measured using commercial ELISA kits following the instructions for each assay.

Calcidiol serum levels were quantified with the human soluble 25-OH Vitamin D ELISA Kit (detection limit of 1.6 ng/mL, Eagle Biosciences^®^, VID31-K01, Amherst, NH, USA). For calcitriol quantification, the human soluble 1,25α(OH)_2_D_3_ ELISA kit (sensitivity < 0:10 pg/mL, CUSABIO^®^, CSB-E0512h, Wuhan, China) was used, and the sVDR quantification was performed with the Human VDR ELISA Kit (Sandwich ELISA) (detection range: 6.25–400 pg/mL); CUSABIO^®^, CSB-E05136h, Wuhan, China). Hs-CRP serum-level quantification was performed with the high-sensitivity CRP (hs-CRP) kit (COD 31927, BioSystems^®^, Barcelona, Spain). The detection limit of the assay was 0.06 mg/L, and the measurement interval was 0.06–15 mg/L. The glucose and lipid profiles (triglycerides, total cholesterol, HDL-C, and LDL-C) were determined with colorimetric enzymatic assays (BioSystems^®^ kits, Barcelona, Spain) using semi-automated equipment (Mindray-BS-240 Clinical Chemistry Analyzer, Shenzhen, China).

### 2.4. Vitamin D Molecules Cutoff Values and CVD Risk Criteria Classification

Calcidiol serum levels were interpreted as deficiency (<20 ng/mL), insufficiency (≥20–<30 ng/mL), and sufficiency (≥30 ng/mL), taking as a reference that reported by Holick et al. in 2007 [22]. The vitamin D hydroxylation efficiency ratio was calculated using the quotient calcitriol (pg)/calcidiol (ng), to establish the amount of calcidiol (ng/mL) being hydroxylated to calcitriol (pg/mL) [23]. The vitamin D hydroxylation efficiency ratio was also analyzed and classified in tertiles per group: RA: T1st or low conversion rate (<1.3 pg/ng), T2nd or average conversion rate (≥1.35–<2.16 pg/ng), and T3rd or high conversion rate (≥2.16 pg/mL). sVDR serum levels were analyzed in each group and categorized into tertiles as follows: the sVDR tertile classification was: T1st or low sVDR levels (<4.67 pg/mL), T2nd or mild sVDR levels (≥4.67–<11.7 pg/mL), and T3rd or high sVDR levels (≥11.7 pg/mL).

CVD risk was evaluated with CRP cutoff values based on the criteria of the Centers for Disease Control and Prevention and the American Heart Association. CRP serum levels for low CVD risk were <3.0 mg/L and those for high CVD risk were ≥3.0 mg/L [9]. Cardiometabolic indexes were also calculated according to formulas described in detail in the study by Campos-López et al. [24]: (a) Castelli index, (b) Kannel index, (c) TG/HDL-C, (d) cardiometabolic index (CMI score), and (e) lipid accumulation products (LAP score).

### 2.5. Statistical Analysis

Statistical analysis was performed using the STATA v 15.0 software (College Station, TX, USA) and GraphPad Prism v 8.0 (San Diego, CA, USA). A normality test was performed with the Shapiro–Wilk test in continuous variables. Nominal discontinuous variables were expressed as frequencies. Descriptive data were expressed as mean ± standard deviation (SD) and medians, with 5th–95th percentiles for parametric and non-parametric variables, respectively. Student’s *t*-tests and Mann–Whitney U tests were performed to assess differences between the two groups. The Kruskal–Wallis test was applied to calculate differences between three groups, and Dunn’s test was used as a post hoc. Categorical variables were compared using the Fisher X^2^ test. A receiver operating characteristic curve (ROC) analysis was performed to determine the discriminative capacity of the sVDR to differentiate between RA and CS; active RA and remission RA patients; RA patients with 0 tender joints and RA patients with ≥1 tender joint; and RA patients with 0 swollen joints and RA patients with ≥1 swollen joint. Cutoff values for area under the ROC curve (AUC) accuracy were: AUC ≤ 0.5 = low discrimination capacity, AUC ≤ 0.8 = acceptable discrimination capacity, AUC > 0.8–0.9 high discrimination capacity, and AUC > 0.9 = excellent discrimination capacity [25]. Spearman’s correlation test was used to calculate the correlations between the sVDR and anthropometric, biochemical, and clinical variables, vitamin D metabolites (calcidiol and calcitriol), and the calcitriol/calcidiol ratio. A *p*-value of <0.05 was considered statistically significant.

## 3. Results

### 3.1. Anthropometric, Biochemical, and Clinical Variables in RA and HS

A total of 154 RA patients and 201 HS were assessed; the general characteristics of both groups are shown in Table 1. RA patients have a higher weight (RA = 67.3 kg vs. HS = 62.5 kg; *p* < 0.01); BMI (RA = 27.48 kg/m^2^ vs. HS = 24.06 kg/m^2^; *p* < 0.001); waist circumference (RA = 87.7 cm vs. HS = 77.7 cm; *p* < 0.001); and WHR than HS (RA = 0.84 vs. HS = 0.77; *p* < 0.001). Additionally, RA patients have higher serum levels of triglycerides (RA = 96.7 mg/dL vs. HS = 76.6 mg/dL; *p* < 0.001) than HS. Regarding clinical variables in RA patients, the mean of disease duration was 6.5 years. Moreover, 36% were in clinical disease remission (DAS 28 ≤ 2.6) and 64% had clinical activity (DAS 28 > 2.6). The median DAS28-CRP score was 3.07 (1.46–5.83), and the median DAS28-ESR score was 3.6 (1.8–6.13). Also, CRP serum levels were higher in RA patients vs. HS (RA = 4.4 (0.5–29.42) mg/L vs. HS = 1.19 (0–12.4) mg/L; *p* < 0.001) (Table 1).

### 3.2. Vitamin D Molecules in RA Patients and HS

**Figure 1:**
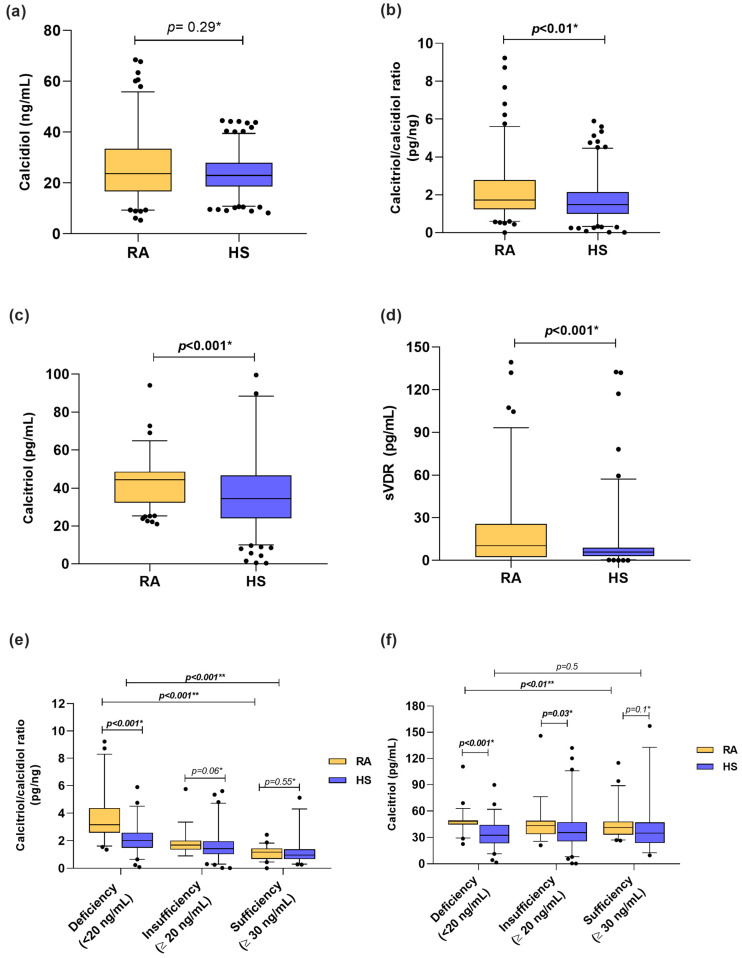
Vitamin D molecules serum levels in RA patients and HS stratified by calcidiol categories. (**a**) Calcidiol serum levels in RA patients vs. HS. (**b**) Calcitriol/calcidiol ratio in RA patients vs. HS. (**c**) Calcitriol serum levels in RA vs. HS. (**d**) sVDR serum levels in RA vs. HS. (**e**) Calcitriol/calcidiol ratio score in RA patients and HS stratified by calcidiol categories. (**f**) Calcitriol serum levels in RA patients and HS stratified by calcidiol categories. Data shown as median and p5th–p95th percentiles. * *p* value: U Mann–Whitney test and ** *p* value Kruskal–Wallis test. sVDR: soluble vitamin D receptor; RA: Rheumatoid arthritis; HS: Healthy subjects.

Among the vitamin D molecules, when we stratified according to calcidiol reference cutoff values, we identified that 37% of RA patients had vitamin D deficiency, 30% had insufficiency, and 33% had sufficient levels vs. 36%, 44%, and 20%, respectively, in HS. In both groups, the median calcidiol serum levels from RA patients and HS were classified as vitamin D insufficiency (RA = 23.71 ng/mL, HS = 22.99 ng/mL) (Figure 1a). Moreover, calcitriol serum levels (RA = 44.35 pg/mL vs. HS = 34.37 pg/mL; *p* < 0.001) and the calcitriol/calcidiol ratio (RA = 1.72 pg/ng; HS = 1.47 pg/ng; *p* < 0.01) were higher in patients with RA than HS (Figure 1b,c). In the case of the sVDR, RA patients had higher concentrations than HS (RA = 10.91 pg/mL vs. 5.9 pg/mL; *p* < 0.001) (Figure 1d). In addition, we analyzed the calcitriol/calcidiol ratio and calcitriol serum levels stratified by calcidiol categories (Figure 1e,f). We observed that RA patients with vitamin D deficiency were hydroxylating more calcidiol to calcitriol (RA = 3.16 pg/ng vs. HS = 2.01 pg/ng; *p* < 0.001) (Figure 1e), which was also reflected in higher serum levels of calcitriol (RA = 48.08 pg/mL vs. HS = 32.48 pg/mL) than HS with vitamin D deficiency (Figure 1f).

### 3.3. Anthropometric, Biochemical, Clinical Variables, and Vitamin D Metabolites Stratified by sVDR Tertiles

HS with the lowest levels of the sVDR had a higher weight, BMI, waist circumference, hip circumference, WHR, and triglycerides serum levels. There was no difference between vitamin D metabolites stratified by sVDR tertiles (Supplementary Table S1). Conversely, RA patients with a higher sVDR level had higher calcitriol, CRP, triglycerides, and LDL-C serum levels, as well as higher WHR scores, than those in T1st and T2nd. The DAS28-ESR score was lower in RA patients with the highest sVDR level, and they had the least number of swollen joints (zero swollen joints) (Supplementary Table S2).

### 3.4. Correlations between Vitamin D Molecules with Anthropometrical, Biochemical, and Clinical Variables in RA Patients and HS

We conducted a correlation analysis between calcidiol, calcitriol, and its hydroxylation efficiency according to calcitriol/calcidiol ratio scores, in both groups. Notably, in both study groups, we observed a negative correlation between calcidiol and the hydroxylation efficiency ratio (RA: r = −0.88, *p* < 0.001; HS: r = −0.46, *p* < 0.001). Conversely, a positive correlation between hydroxylation efficiency ratio with calcitriol serum levels was observed in RA and HS (RA: r = 0.69, *p <* 0.001; HS: r = 0.80, *p* < 0.001).

**Figure 2:**
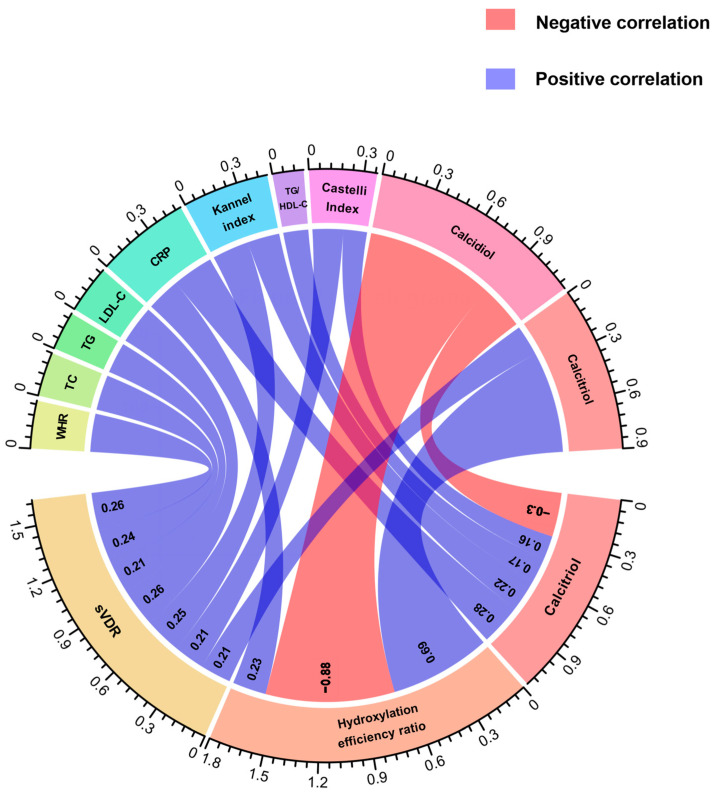
Correlations of anthropometrical, biochemical, and cardiometabolic variables and vitamin D metabolites with the sVDR, vitamin D hydroxylation efficiency ratio, and calcitriol serum levels in RA patients. Spearman correlation coefficients are represented as connecting lines between the anthropometrical, biochemical, cardiometabolic variables, and vitamin D metabolites (top rainbow) and the sVDR, calcitriol/calcidiol ratio, and calcitriol (bottom rainbow). Only significant correlations with a *p*-value < 0.05 are shown.. The width of lines represents the value of the correlation coefficients (measured with scale). For all the correlation analyses, see Supplementary Table S3. sVDR: soluble vitamin D receptor; Ratio: Hydroxylation efficiency ratio; WHR: Waist-to-hip ratio; TC: Total cholesterol; TG: Triglycerides; LDL-C: Low-density lipoprotein cholesterol; CRP: C-reactive protein; TG/HDL-C: Triglycerides/HDL-C index.

Regarding this analysis, in RA patients, we observed positive correlations between the sVDR and WHR (r = 0.26, *p =* 0.01), total cholesterol (r = 0.24, *p* = 0.01), triglycerides (r = 0.21, *p =* 0.03), LDL-C (r = 0.26, *p <* 0.01), Kannel index (r = 0.25, *p* = 0.01) and calcitriol (r = 0.21, *p =* 0.03) (Figure 2). In the case of the hydroxylation efficiency ratio score, it only correlated positively with CRP in RA patients (r = 0.23, *p* = 0.01) (Figure 2). According to calcitriol, RA patients had a positive correlation between calcitriol serum levels and CRP (r = 0.28, *p* < 0.001), Kannel index score (r = 0.22, *p* <0.01), TG/HDL-C index score (r = 0.17, *p =* 0.04), and Castelli index score (r = 0.16, *p* = 0.04), and a negative correlation with calcidiol serum levels (r = −0.30, *p* < 0.001 (Figure 2).

Conversely, in HS, we found a negative correlation between the sVDR and age (r = −0.29, *p* < 0.01), weight (r = −0.26, *p* < 0.01), BMI (r = −0.25, *p* < 0.01), waist circumference (r = −0.30, *p* = 0.001), hip circumference (r = −0.27, *p*< 0.01), WHR (r = −0.21, *p* = 0.02), triglycerides (r = −0.25, *p* < 0.01), and TG/HDL-C index (r = −0.22, *p* = 0.02) (Supplementary Table S3).

### 3.5. Vitamin D Molecules Stratified by CVD Risk and Clinical Variables

**Figure 3:**
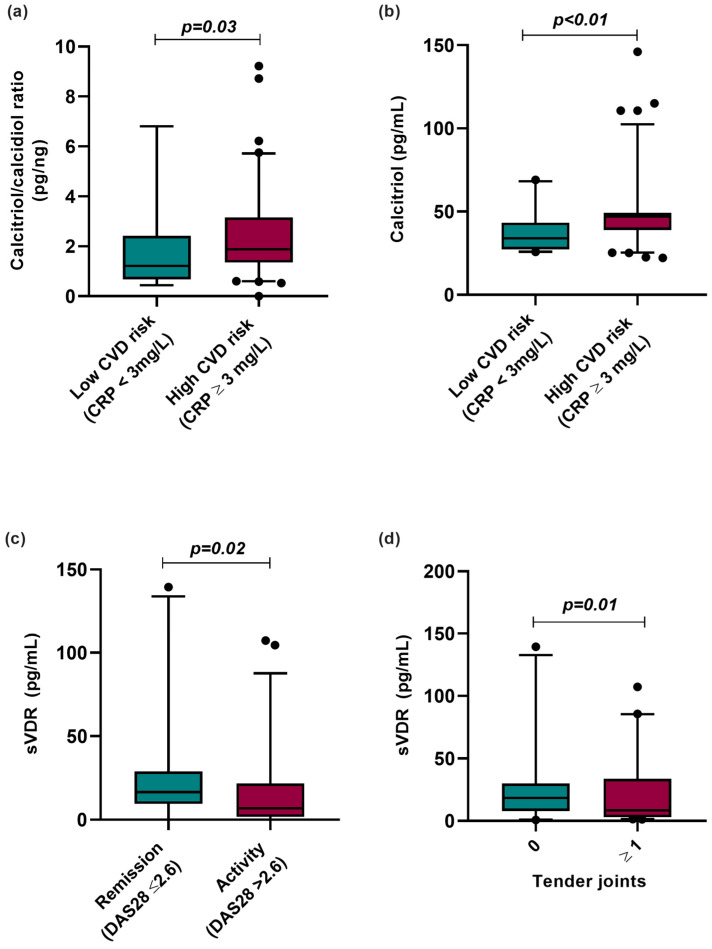
Vitamin D molecules stratified according to CVD risk by CRP and clinical variables in RA patients. (**a**) Calcitriol/calcidiol ratio stratified by CVD risk using CRP serum levels (>3 mg/L). (**b**) Calcitriol serum levels stratified by CVD risk using CRP serum levels (≥3 mg/L). (**c**) sVDR level stratified by clinical activity and remission in RA patients. (**d**) sVDR level stratified by number of tender joints in RA patients. Data are shown as median and p5th–p95th percentile. *p*-value: Mann–Whitney U test. CRP: C-reactive protein; sVDR: Soluble vitamin D receptor; RA: Rheumatoid arthritis; HS: Healthy subjects; DAS28: Disease Activity Score 28 joints.

We analyzed the vitamin D metabolites according to CVD risk categorized by CRP serum levels, clinical disease activity, and clinical variables such as the number of tender joints in RA patients (Figure 3). According to this analysis, RA patients with CRP ≥ 3 mg/L, considered high risk for CVD, had a higher hydroxylation ratio (CRP < 3 mg/L = 1.12 pg/ng vs. CRP ≥ 3 mg/L = 1.88 pg/ng; *p =* 0.003) and higher calcitriol serum levels (CRP < 3 mg/L = 33.93 pg/mL vs. CRP > 3 mg/L = 47.1 pg/mL; *p <* 0.01) than RA patients with CRP levels lower than 3 mg/L, considered low risk for CVD (Figure 3a,b).

Moreover, we analyzed sVDR levels by clinical activity, observing that RA patients in clinical remission (remission: 18.32 pg/mL vs. activity: 7.01 pg/mL; *p* = 0.02) and with the absence of tender joints (0 tender joints: 18.44 pg/mL vs. ≥1 tender joints: 7.63 pg/mL; *p* = 0.01) had higher sVDR levels than RA patients with clinical disease activity and the presence of tender joints (Figure 3c,d).

**Figure 4:**
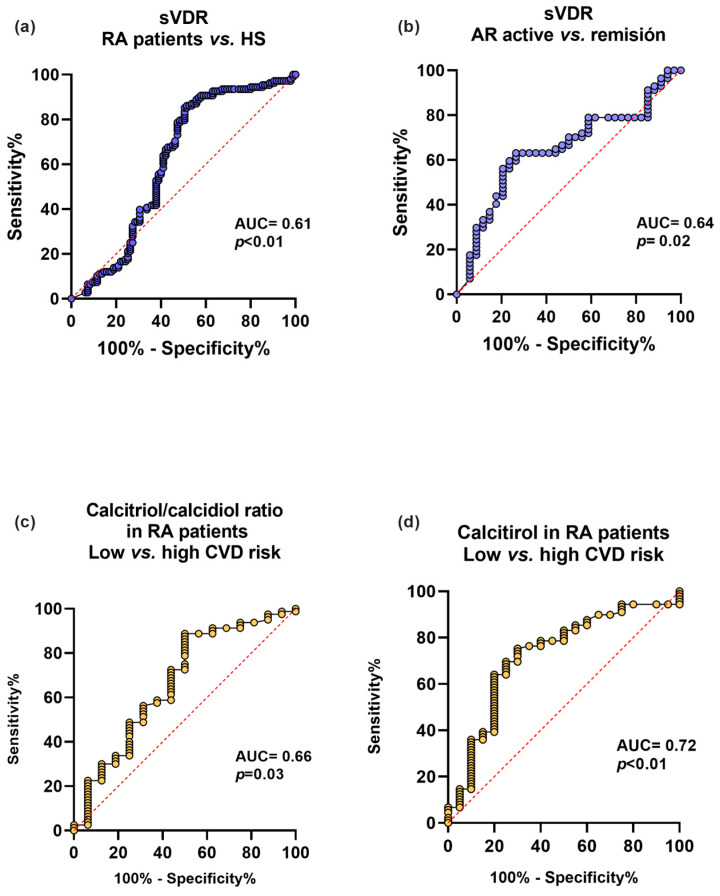
Discriminatory capacity of the sVDR, calcitriol, and its hydroxylation efficiency ratio for the disease, clinical activity, and CVD in RA patients. (**a**) Discriminatory ROC curve between RA patients and HS. (**b**) Discriminatory ROC curve for active vs. remission disease activity in RA patients. (**c**) Calcitriol/calcidiol ratio discriminatory ROC curve for low CVD disease risk (<3 mg/L) and high CVD risk (≥3 mg/L) in RA patients. (**d**) Calcitriol discriminatory ROC curve for low CVD risk (<3 mg/L) and high CVD risk (≥3 mg/L) in RA patients. Blue lines: ROC curves with sVDR. Yellow lines: ROC curves with calcitriol/calcidiol ratio and calcitriol. sVDR: soluble vitamin D receptor. AUC = area under the curve.

We analyzed the sVDR capacity to discriminate between RA and HS, and clinical disease activity and remission. We also examined the discriminatory capacity for CVD risk using CRP levels ≥ 3 mg/L of the calcitriol and its hydroxylation efficiency ratio in RA patients (Figure 4). First, the sVDR had an acceptable capacity to discriminate between RA and HS (AUC = 0.61, *p <* 0.01) (Figure 4a) and to discriminate between clinical disease activity and remission (AUC = 0.64, *p* = 0.02) (Figure 4b). Additionally, the calcitriol/calcidiol hydroxylation efficiency ratio had a moderate discriminatory capacity for CVD risk in RA patients (AUC = 0.66, *p* = 0.03) (Figure 4c), and calcitriol was also a moderate discriminator for CVD risk in RA patients (AUC = 0.72, *p* < 0.01) (Figure 4d).

### 3.6. Associations of Potential Vitamin D Molecules Biomarkers with RA Disease and CVD Risk in RA Patients

To estimate the association between vitamin D molecules (calcidiol, calcitriol/calcidiol ratio, calcitriol, and the sVDR) and RA disease and CVD risk using CRP serum levels, we constructed multiple regression models (Table 2). We observed that RA patients had a greater probability of a high calcitriol/calcidiol hydroxylation efficiency ratio score (OR = 2.02; CI: 1.09–3.75; *p* = 0.02), higher calcitriol serum levels (OR = 2.95; CI: 1.62–5.39; *p* < 0.001), and higher sVDR serum levels (OR = 5.57; CI: 2.23–13.9; *p* < 0.001) than HS. This same pattern was also observed using CRP serum levels in RA patients with high CVD risk, who had a higher probability of a higher calcitriol/calcidiol hydroxylation efficiency ratio score (OR = 4.51; CI: 1.09–18.6; *p* = 0.04). This reflects in a higher probability to present mild (OR = 4.22; CI 1.09–14.70; *p* = 0.04) and higher calcitriol levels (OR = 5.6; CI: 1.49–21; *p* < 0.01) (Table 2).

According to these findings, we constructed multiple regression models (β coefficient), and we observed that having RA increases the calcidiol concentration by 2.84 ng/mL (β coefficient = 2.84; *p* = 0.03). Likewise, we observed that a higher CVD risk in RA patients increases calcitriol serum levels by 11.56 pg/mL (β coefficient = 11.56; *p* = 0.01) (Table 3).

## 4. Discussion

RA is a multifactorial autoimmune inflammatory disease. Genetic and environmental risk factors have been associated with the development of this condition [1]. Among the environmental factors, a negative association has been described between alterations in concentrations of vitamin D metabolites, such as hypovitaminosis D. This has been negatively associated with clinical disease activity and with comorbidities development [26,27]. However, other vitamin D molecules could be implicated in the pathophysiology and severity of the disease, as well as the development of its major comorbidities, such as CVD. In our study, vitamin D deficiency was less frequent in RA patients compared to other populations, but they had a pattern of hypovitaminosis D, with higher serum levels of calcitriol, a higher calcitriol/calcidiol hydroxylation efficiency ratio score, and higher sVDR levels. A similar pattern was also observed in another study in our population by Meza-Meza et al. in SLE patients, where this pattern was associated with a higher disease risk [13]. The elevation of serum calcitriol levels in situations of hypovitaminosis D could be linked to the synthesis of calcitriol by extrarenal tissues, such as immune system cells that maintain physiological functions [22]. High calcitriol levels in pathological conditions may indicate an ongoing inflammatory process. For example, in other conditions such as granulomatous disease and pulmonary tuberculosis, high levels of calcitriol have been associated with worse disease activity. In contexts like cancer, these elevated calcitriol levels promote immunosuppression in the anti-tumoral microenvironment due to their anti-inflammatory properties, which could favor tumor progression [28,29].

Moreover, serum levels of sVDR were also higher in patients with RA compared to HS. VDR is a ligand-dependent transcription factor receptor located in the nucleus and cytoplasm [30,31]. Notably, due to its intracellular location, it should not or would not be expected to be present in the bloodstream. However, a few studies in different diseases have reported its presence in serum and different concentrations compared to healthy subjects. A study in patients with colorectal cancer reports a decrease in serum levels of VDR compared to control subjects (0.49 ± 0.13 ng/mL vs. 4.08 ± 0.43 ng/mL, *p* < 0.0001) [14]. Also, in children with autism spectrum disorders, lower levels of the sVDR were observed in comparison to the control group [15]. Studies in other diseases such as irritable bowel syndrome and gestational diabetes mellitus reported higher sVDR levels in patients than the control group (*p* = 0.001, (*p* < 0.001, respectively) [16,17]. We only found one study in other autoimmune disease. In 2019, Kültür, T., et al. analyzed the association between the sVDR and disease activity and clinical parameters in patients with ankylosing spondylitis (AS). They observed that sVDR levels were higher in patients with active AS (BASDAI score ≥ 4, *p* = 0.01), as well as a positive correlation between the sVDR and the clinical activity index BASDAI, CRP, and ESR in AS patients (*p* = 0.01, r = 0.751; *p* = 0.01, r = 0.75; *p* = 0.01, r = 0.81, respectively). Therefore, they proposed that the sVDR could be a marker of inflammation, along with other molecules such as CRP [18].

CRP is an acute-phase reactant expressed in response to proinflammatory cytokines such as IL-6. It is used as a marker to assess inflammatory processes and the risk of developing CVD [32]. RA patients have a two-fold or greater risk of death from CVD. This increased risk could be attributed to factors such as dyslipidemia, which occurs in up to 60% of RA patients; the presence of diabetes mellitus; high BMI; and the presence of hypertension. Likewise, the pro-inflammatory environment present in RA itself increases CVD risk [33,34]. Regarding this, we decided to perform correlation analyses of vitamin D molecules with biochemical and cardiometabolic variables. Notably, we observed a positive correlation between the calcitriol/calcidiol hydroxylation efficiency ratio score with CRP, and calcitriol serum levels positively correlated with CRP, the Kannel index, TG/HDL-C ratio, and the Castelli index. This suggests that in RA patients, as calcitriol levels increase, so does the CVD risk. However, this differs from what has been previously described in other studies, where a negative correlation has been observed between serum levels of calcitriol and inflammatory and cardiovascular variables, such as CRP, TNF-α, uric acid, homocysteine, and fibrinogen. Additionally, a higher risk of coronary artery disease, hypertension, and heart failure has been documented in patients with low levels of calcitriol [35,36]. It is worth noting that none of these studies were conducted in RA patients or those with any other autoimmune disease. On the other hand, in vitro studies have described that calcitriol has a dual dose-dependent effect. For example, in vitro studies on chondrocytes and keratinocytes have demonstrated that the ability of calcitriol to stimulate or inhibit cell proliferation is linked to its concentration gradient; at higher concentrations, cellular proliferation decreases [37]. It has also been observed that calcitriol could regulate other growth factors such as endothelin in cardiomyocytes in an in vivo model [38]. Endothelin is a potent vasoconstrictor that has been associated with increased mortality in patients with and without CVD [39]. On the other hand, it is well-described that having low levels of calcidiol increases mortality and CVD risk, as well as the concentration of systemic inflammatory markers [35,40]. Additionally, Pasquali et al. observed in 2015 that in subjects with hypovitaminosis D, there is an increase in the hydroxylation efficiency ratio assessed using the calcitriol/calcidiol ratio score, and it was shown in high calcitriol serum levels. This pattern was also observed in SLE patients, where a pattern of higher calcitriol serum levels was associated with a higher risk for the disease [13,23]. Notably, we observed this same pattern in our RA patients: we noted using CRP serum levels that patients with a high CVD risk exhibit a higher calcitriol/calcidiol hydroxylation efficiency ratio and calcitriol levels compared to those with a lower CVD risk determined by CRP. Likewise, we observed that in RA patients, both the hydroxylation ratio and calcitriol are biomarkers with a moderate capacity to discriminate between low and high CVD risk. Furthermore, we found an association between CRP level ≥ 3 mg/L and an increase in calcitriol serum levels and its hydroxylation efficiency ratio score.

Regarding sVDR levels, we also observed its capacity to discriminate between RA patients and HS, as well as between RA patients in remission and those with clinical disease activity according to the DAS28 index. This capacity of the sVDR to discriminate according to clinical activity had previously been observed in AS patients; in this study, a positive correlation was found between clinical disease activity and acute-phase reactants, such as CRP and ESR, and the sVDR [18]. Moreover, in vitro studies have described that in the presence of proinflammatory cytokines such as TNF-α, the expression of VDR increases, correlating positively with the expression of IL-6. It is worth noting that IL-6 stimulates the expression of CRP in hepatocytes. Therefore, it has been suggested that the sVDR in serum could be a potential biomarker of inflammation or an acute-phase reactant, which could be used to assess the clinical activity of autoimmune diseases [41,42,43]. However, the mechanisms that support this hypothesis remain unknown.

Considering the above, we propose that molecules from vitamin D metabolism, such as high calcitriol, its hydroxylation efficiency ratio score, and the sVDR in conjunction with high CRP concentrations, could support the existing biomarkers of inflammation and CVD risk in RA patients. However, further investigations are needed to evaluate the potential risks and patterns over time of these molecules in RA and other autoimmune diseases. Some limitations of this study include the omission of genetic variables that may impact key molecules in vitamin D metabolism and VDR expression, as well as the non-quantification of other molecules influencing vitamin D concentrations, such as PTH, cholecalciferol, vitamin D binding protein, and calcium serum levels. It is noteworthy that this study is the first exploration that analyzes serum levels of calcitriol, its hydroxylation efficiency ratio, and the sVDR to assess their potential associations with clinical disease activity and CVD risk in Mexican RA patients.

## 5. Conclusions

Hypovitaminosis D in RA patients was characterized by a pattern of a higher hydroxylation efficiency ratio and higher calcitriol and sVDR serum levels. Notably, higher calcitriol serum levels and its vitamin D hydroxylation efficiency ratio were associated with higher CVD risk in RA patients.

## Figures and Tables

**Table 1:** Anthropometric, biochemical, and clinical variables in RA patients and HS.

Variable	RA Patients (*n* = 154)	HS (*n* = 201)	*p*-Value
Age (years) ^a^	35 (19–59)	48 (27–65)	**<0.001**
Height(cm) ^b^	1.57 ± 0.05	1.61 ± 0.06	**<0.01**
Weight (kg) ^a^	67.3 (48.9–93.4)	62.5 (47.7–86.7)	**<0.01**
BMI (kg/m^2^) ^a^	27.48 (19.78–36.27)	24.06 (18.75–34.89)	**<0.001**
Waist circumference (cm) ^a^	87.7 (69–108)	77.7 (61.5–105)	**<0.001**
Hip circumference (cm) ^a^	103.5 (88–127)	100 (88.2–119.2)	**<0.01**
WHR ^a^	0.84 (0.74–0.98)	0.77 (0.68–0.93)	**<0.001**
Glucose (mg/dL) ^a^	88 (72.58–127)	88.44 (75.14–114.23)	0.24
Albumin (g/dL) ^a^	3.91 (3.25–4.48)	3.81 (3.39–4.42)	**<0.05**
Triglycerides (mg/dL) ^a^	96.72 (44.65–195)	76.63 (39.41–198.72)	**<0.001**
Cholesterol (mg/dL) ^a^	169.5 (114–237)	174.46 (125.25–243.82)	0.17
HDL-C (mg/dL) ^a^	49.2 (30.1–76.24)	51.34 (35.28–75.93)	0.07
LDL-C (mg/dL) ^a^	94.39 (55.37–147.14)	95.23 (61.61–157.25)	0.22
Disease duration (years) ^a^	6.5 (1–22)		
DAS28 (CRP) ^a^	3.07 (1.46–5.83)	-	-
DAS28 (ESR) ^b^	3.7 ± 1.3	-	-
Tender joints ^a^	0.5 (0–10)	-	-
Swollen joints ^a^	1.5 (0–12)	-	-
Remission (DAS28-CRP ≤ 2.6) ^c^	36 (51/142)	-	-
Activity (DAS28-CRP > 2.6) ^c^	64 (91/142)	-	-
Hs-CRP (mg/L) ^a^	4.4 (0.5–29.42)	1.19 (0–12.4)	**<0.001**
ESR (mm/h) ^a^	35.5 (8–79)	-	-
ACPAs (UI/mL) ^a^	208 (13.4–289)	-	-
RF (UI/mL) ^a^	114 (11–500)	-	-

^a^ Data provided as median (percentile: p5th–p95th), *p*-value: U Mann–Whitney test. ^b^ Data provided as median ± SD, *p*-value: Student’s *t*-test. ^c^ Data provided as % (*n*), *p*-value: F-fisher. Bold numbers indicate significant differences. (-): Indicates no data. BMI: Body mass index; WHR: Waist-to-hip ratio; HDL-C: High-density lipoprotein; LDL: Low-density lipoprotein; DAS: Disease Activity Score; ESR: Erythrocyte sedimentation rate; Hs-CRP: High-sensitivity C-reactive protein; ACPAs: Anti-citrullinated peptide antibodies; RF: Rheumatoid factor.

**Table 2:** Associations of potential vitamin D molecules biomarkers with RA disease and CVD risk.

Vitamin D Molecules			RA Patients vs. HS				CVD Risk (CRP ≥ 3 mg/L)			
			Without Adjusted		Multiple Models ^∞^		Without Adjusted		Multiple Models ^∞^	
	% (*n*)		OR (CI 95%)	*p*-Value	OR (CI 95%)	*p*-Value	OR (CI 95%)	*p*-Value	OR (CI 95%)	*p*-Value
Calcidiol (ng/mL)	Deficiency *	36 (36)	1	-	1	-	1	-	1	-
	Insufficiency	15 (15)	0.65 (0.38–0.79)	0.11	0.73 (0.41–1.3)	0.29	0.7 (0.18–2.77)	0.6	0.8 (0.21–3.36)	0.8
	Sufficiency	48 (48)	1.63 (0.92–2.87)	0.09	1.6 (0.91–3.13)	0.09	0.67 (0.18–2.47)	0.55	0.8 (0.21–3.39)	0.8
Calcitriol/calcidiol ratio (pg/ng)	Low *	26 (34)	1	-	1	-	1	-	1	-
	Mild	34 (43)	1.28 (0.73–2.26)	0.38	1.76 (0.95–3.31)	0.07	3.37 (0.87–13.02)	0.07	3.58 (0.86–14.9)	0.08
	High	40 (51)	1.90 (1.08–3.35)	**0.02**	2.02 (1.09–3.75)	**0.02**	4.62 (1.22–17.6)	**0.03**	4.51 (1.09–18.6)	**0.04**
Calcitriol (pg/mL)	Low *	35 (50)	1	-	1	-	1	-	1	-
	Mild	30 (43)	2.4 (1.18–3.5)	**0.01**	2.68 (1.45–4.98)	**<0.01**	3.9 (1.08–14.14)	**0.04**	4.02 (1.09–14.7)	**0.04**
	High	35 (51)	2.73 (1.59–4.69)	**<0.001**	2.95 (1.62–5.39)	**<0.001**	5.8 (1.63–20.6)	**<0.01**	5.6 (1.49–21)	**0.01**
sVDR (pg/mL)	Low *	36 (36)	1	-	1	-	1	-	1	-
	Mild	15 (15)	0.4 (0.18–0.79)	**<0.01**	0.98 (0.38–2.48)	0.97	-	-	-	-
	High	48 (48)	3.75 (1.83–7.7)	**<0.001**	5.57 (2.23–13.9)	**<0.001**	5.3 (0.93–30.1)	0.06	8.43 (1.08–65.4)	0.04

RA: Rheumatoid arthritis; Low: tertile 1; Mild: tertile 2; High: tertile 3. ∞ Adjusted by age (years), body mass index (BMI), fat mass (%). OR = Odds ratio of generalized estimation equation (GEE). CI 95%, Confidence interval of 95%. (-): indicates no data. Bold numbers indicate significant differences. * Reference category.

**Table 3:** Effect of RA disease and CVD risk using CRP serum levels on vitamin D molecules.

	Without Adjusted		Multiple Models ^1^	
	^◊^ β (95% CI)	*p*-Value *	β (95% CI)	*p*-Value *
RA disease				
Calcidiol (ng/mL)	2.89 (0.56–5.23)	**0.01**	2.84 (0.32–5.37)	**0.03**
Calcitriol/calcidiol ratio (pg/ng)	0.45 (0.04–0.85)	**0.03**	0.31 (−0.11–0.75)	0.15
Calcitriol (pg/mL)	2.53 (−5.8–10.87)	0.55	−0.62 (−9.73–8.49)	0.89
sVDR (pg/mL)	14.39 (−27.06–55.86)	0.49	29.77 (−22.89–82.44)	0.26
CVD risk (CRP ≥ 3 mg/L)				
Calcidiol (ng/mL)	−5.9 (−13.5–1.66)	0.12	−6.1 (−13.6–1.45)	0.11
Calcitriol/calcidiol ratio (pg/ng)	0.69 (−0.21–1.59)	0.13	0.71 (−0.20–1.63)	0.13
Calcitriol (pg/mL)	10.74 (1.84–19.65)	**0.02**	11.56 (2.56–20.55)	**0.01**
sVDR (pg/mL)	35.34 (−71.7–142.47)	0.51	47.69 (−64.55–159.94)	0.39

◊: Regression beta coefficient (95% CI); ^1^ Adjusted by BMI (kg/m^2^) and age (years). *p* * Model’s generalized regression. Bold numbers indicate significant difference. CI: Confidence interval; sVDR: Soluble vitamin D receptor; BMI: Body mass index. RA: Rheumatoid arthritis; CVD: Cardiovascular disease; CRP: High-sensitivity C-reactive protein.

## Data Availability

Data used to support the findings of this study are available from the corresponding author upon reasonable request.

## Supplementary Materials

The following supporting information can be downloaded at: https://www.mdpi.com/article/10.3390/biomedicines12020273/s1, Table S1: Anthropometric, biochemical, clinical variables and vitamin D metabolites stratified by sVDR tertiles in HS, Table S2: Anthropometric, biochemical, clinical variables and vitamin D metabolites stratified by sVDR tertiles in RA patients, Table S3: Correlations between the sVDR and anthropometrical, biochemical, and clinical variables and vitamin D metabolites in RA patients and HS, Table S4. Anthropometric, biochemical, and clinical variables in RA patients by calcidiol classification.
