# Supplementary material for: Analysis of Potential Vitamin D Molecule Biomarkers: Association of Calcitriol and Its Hydroxylation Efficiency Ratio with Cardiovascular Disease Risk in Rheumatoid Arthritis Patients
Source: Biomedicines. 2024 Jan 25;12(2):273. doi: 10.3390/biomedicines12020273 (PMC10886495; doi:10.3390/biomedicines12020273)
Supplement: Supplementary file 1 [file biomedicines-12-00273-s001.zip › biomedicines-2812628-supplementary.pdf]

Supplementary Table S1. Anthropometric, biochemical, clinical variables and vitamin D metabolites stratified by sVDR tertiles in healthy subjects (HS)

Healthy Subjects (CS)	sVDR (pg/mL)			
	**T1: 0.72 – 4.67 (pg/mL) n=45**	**T2: 4.68 – 11.7 (pg/mL) n=49**	**T3: 11.71 – 367 (pg/mL) n=16**	***p* value***
Anthropometric				
**Age (years)[a]**	**31 (19 - 56)**	**23 (19 – 61)**	**22 (19 – 61)**	**0.01**
Height(cm)[a]	1.62 (1.54 – 1.72)	1.6 (1.53 – 1.71)	1.62 (1.53 – 1.76)	0.46
**Weight (kg)[a]**	**64.15 (47.7 – 82.9)**	**55.4 (46.1 – 70.1)**	**59.05 (43.6 – 65.4)**	**0.01**
**BMI (kg/m2)[a]**	**23.6 (18.6 – 30.8)**	**22.1 (18.2 – 27.7)**	**22.1 (18.6 – 24.7)**	**0.01**
**Waist circumference (cm)[a]**	**76.3 (62.5 – 100.1)**	**71 (62– 88.5)**	**69.3 (61.3 – 79)**	**<0.01**
**Hip (cm)[a]**	**101 (88.5 – 116)**	**95 (85.5 – 104.7)**	**96 (86.4 – 102)**	**<0.01**
**WHR [a]**	**0.77 (0.67 – 0.89)**	**0.74 (0.67 – 0.89)**	**0.71 (0.66 – 0.79)**	**<0.05**
Biochemicals				
Glucose (mg/dL)[a]	84.65 (75.2 – 103.1)	83.8 (72.9 – 100.7)	83.7 (76.8 – 113.9)	0.36
**Albumine (g/dL)[a]**	**3.67 (3.47 – 4.21)**	**3.89 (3.51 – 4.42)**	**3.76 (3.51 – 4.21)**	**<0.01**
**Triglycerides (mg/dL)[a]**	**74.76 (36 – 145.3)**	**64.11 (37.02 - 130.63)**	**53.23 (39.41 – 118.75)**	**<0.05**
Cholesterol (mg/dL)[a]	165.41 (118.84 – 233.1)	157.29 (120.68 – 228.69)	158.06 (100.43 – 245.53)	0.38
HDL-C (mg/dL)[a]	50.32 (27.7 – 71.54)	54.5 (39.97 – 70.66)	54.09 (37.51 – 73.54)	0.26
LDL-C (mg/dL)[a]	94.91(59.14 – 147.27)	90.11 (58.57 – 157.98)	93.87 (49.57 – 170.58)	0.60
Vitamin D metabolites				
**Calcidiol (ng/mL)[a]**	22.01 (12.24 – 40.44)	23.89 (15.48 – 40.28)	24 (14.51 – 44.57)	0.30
**Calcitriol (pg/mL)[a]**	33.32 (12.5 – 82.58)	40.04 (9.64 – 54.28)	38.3 (17.58 – 51.5)	0.48

[a]Data provided in median (percentile: p5th–p95th), *p* value*: Kruskall Wallis test*. sVDR: soluble vitamin D receptor; BMI: Body mass index; WHR: Waist to hip ratio; HDL-C: High density lipoprotein; LDL: Low density lipoprotein.

Supplementary Table S2. Anthropometric, biochemical, clinical variables and vitamin D metabolites stratified by sVDR tertiles in rheumatoid arthritis (RA)

Rheumatoid Arthritis (RA)	sVDR (pg/mL)			
	T1: 0.72 – 4.67 (pg/mL) (n=36)	T2: 4.68 – 11.7 (pg/mL) (n=15)	T3: 11.71 – 367 (pg/mL) (n=48)	*p* value*
Anthropometric				
**Age (years)[a]**	**45.5 (28 – 67)**	**42 (22 – 57)**	**48 (34 – 61)**	**0.04**
Height(cm)[a]	1.56 (1.48 – 1.66)	1.6 (1.48 – 1.72)	1.56 (1.48 – 1.66)	0.24
Weight (kg)[a]	69 (48.2 – 97.2)	68.1 (51.6 – 104)	66 (51.6 – 88.3)	0.71
BMI (kg/m2)[a]	27.4 (19.8 – 36.6)	26.9 (19.7 – 39.6)	26.8 (20.1– 34.5)	0.86
Waist (cm)[a]	86 (68 – 116.4)	89 (74 – 122)	91 (69 – 104)	0.43
Hip (cm)[a]	105.3 (89.1 – 131)	102 (95.5 – 139.5)	103 (91.5 – 118.5)	0.82
WHR [a]	**0.8 (0.7 – 0.9)**	**0.9 (0.7 – 1.01)**	**0.9 (0.7 – 0.9)**	**0.03**
Biochemicals				
Glucose (mg/dL)[a]	**86 (76 – 119)**	**88 (72.58 – 120)**	**90.46 (78.62 – 127)**	**0.10**
Albumine (g/dL)[a]	3.88 (3.33 – 4.63)	3.97 (3.2 – 4.49)	3.95 (3.15 – 4.46)	0.99
Triglycerides (mg/dL)[a]	**80.96 (49.56 – 156)**	**76.69 (28.7 – 151)**	**110.27 (63.81 – 195)**	**0.015**
Cholesterol (mg/dL)[a]	160. 29 (119.23 – 244.21)	173 (81.3 – 217.5)	177 (123 – 247)	0.11
HDL-C (mg/dL)[a]	49.48 (32.63 – 76.24)	45.64 (15.57 – 65.46)	44.34 (28.99 – 77.45)	0.61
LDL-C (mg/dL)[a]	**83.41 (42 – 172)**	**100.25 (52.35 – 133.65)**	**102.92 (73.19 – 151.37)**	**0.02**
Vitamin D metabolites				
Calcidiol (ng/mL)[a]	24.32 (10.16 – 44.81)	20.69 (8.94 – 37.45)	22.21 (9.35 – 37.4)	0.21
Calcitriol (pg/mL)[a]	**44.95 (29.88 – 69.07)**	**45.41 (22.56 – 50.79)**	**48.08 (35.79 – 72.75)**	
Clinical variables				
Time of evolution (years) [a]	6.5 (1 – 25)	3 (0.5 – 13)	7.5 (1 – 22)	0.11
DAS 28 (CRP) [a]	3.2 (1.74 – 5.46)	2.84 (1.12 – 5.9)	2.5 (1.33 – 5.85)	0.05
DAS 28 (ESR) [a]	**4.32 (2.42 – 6.34)**	**3.29 (1.65 – 4.97)**	**2.64 (1.47 – 5.29)**	**<0.01**
Tender joints[a]	2 (0 - 5)	0 (0 – 6)	0 (0 – 7)	0.11
Swollen joints [a]	**3 (0 – 12)**	**2 (0 – 8)**	**1 (0 – 7)**	**0.01**
ESR (mm/hour)[a]	41 (15 – 63)	35 (10 – 48)	22 (7 – 58)	0.13
CRP (mg/dL)[a]	**2.74 (0.5 – 33.79)**	**5.6 (2.3 – 87.7)**	**6 (1.3 – 33)**	**0.01**
Rheumatoid factor (UI/mL)[a]	51.1 (11 – 2560)	105 (48.4 – 156.8)	101.5 (9.6 -177)	0.96
ACPAs (U/mL) [b]	232 (67 – 549)	223 (20.98 – 493.5)	62 (1 – 200)	0.21

[a]Data expressed in median (p5-p95). **Kruskall-Wallis* test. sVDR: soluble vitamin D receptor; BMI: Body mass index; WHR: Waist to hip ratio; HDL-C: High density lipoprotein; LDL: Low density lipoprotein; DAS: Disease activity score; ESR: erythrocyte sedimentation rate; CRP: C-reactive protein; ACPAs: Anti-citrullinated peptide antibodies.

Supplementary Table S3. Correlations between sVDR, calcitriol/calcidiol ratio and calcitriol levels with anthropometrical, biochemical, clinical variables and vitamin D metabolites in rheumatoid arthritis patients (RA) and control subjects (CS).

	**sVDR (pg/mL)**				**Calcitriol/calcidiol ratio (pg/ng)**				**Calcitriol (pg/mL)**			
	**AR (n=99)**		**CS (n=110)**		**AR (n=99)**		**CS (n=110)**		**AR (n=99)**		**CS (n=110)**	
**Variable**	**r**	***p value***	**r**	***p value***	***r***	***p value***	***r***	***p value***	***r***	***p value***	***r***	***p value***
**Age (years)**	0.07	0.47	**-0.29**	**<0.01**	-0.07	0.42	0.03	0.73	-0.06	0.42	-0.07	0.35
**Weight (Kg)**	-0.08	0.39	**-0.25**	**<0.01**	0.001	0.96	0.09	0.2	-0.03	0.72	-0.01	0.86
**BMI (Kg/m²)**	-0.06	0.50	**-0.25**	**<0.01**	-0.02	0.75	0.12	0.11	-0.04	0.60	-0.01	0.88
**Waist circumference (cm)**	0.12	0.23	**-0.30**	**<0.01**	0.06	0.53	0.09	0.22	0.11	0.19	-0.05	0.51
**Hip circumference (cm)**	-0.08	0.44	**-0.27**	**<0.01**	0.04	0.63	0.05	0.48	0.10	0.23	-0.05	0.49
**WHR**	**0.26**	**0.01**	**-0.21**	**0.02**	0.09	0.33	0.09	0.18	0.12	0.15	-0.01	0.88
**Glucosa (mg/dL)**	0.17	0.09	-0.16	0.07	0.09	0.28	0.12	0.09	0.07	0.37	0.05	0.54
**Cholesterol, Total (mg/dL)**	**0.24**	**0.01**	-0.14	0.12	0.19	0.83	-0.01	0.86	0.05	0.49	-0.04	0.57
**Triglycerides (mg/dL)**	**0.21**	**0.03**	**-0.24**	**<0.01**	0.02	0.81	0.08	0.25	0.1	0.19	-0.004	0.95
**HDL-C (mg/dL)**	-0.03	0.70	0.14	0.13	-0.04	0.61	-0.03	0.64	0.08	0.34	0.08	0.22
**LDL-C (mg/dL)**	**0.26**	**<0.01**	-0.09	0.33	0.11	0.22	-0.07	0.36	0.15	0.07	-0.07	0.32
**CRP (mg/L)**	0.20	0.05	-0.13	0.16	**0.23**	**0.01**	0.10	0.17	**0.28**	**<0.001**	0.07	0.35
**Kannel index**	**0.25**	**0.01**	-0.14	0.13	0.13	0.15	-0.07	0.37	**0.22**	**0.007**	-0.13	0.06
**TG/HDL-C index**	0.18	0.06	**-0.22**	**0.02**	0.05	0.54	0.07	0.35	**0.17**	**0.04**	-0.04	0.59
**Castelli index**	**0.21**	**0.03**	-0.18	0.05	0.06	0.47	-0.002	0.97	**0.16**	**0.04**	-0.13	0.08
**Calcidiol (ng/mL)**	-0.13	0.19	0.12	0.21	**-**	**-**	-	-	**-0.3**	**<0.001**	0.08	0.27
**Calcitriol (pg/dL)**	**0.21**	**0.03**	0.10	0.26	**-**	**-**	-	-	-	-	-	-
**Calcitriol/calcidiol ratio (pg/ng)**	0.19	0.05	0.01	0.85	-	-	-	-	-	-	-	-
**Albumine (g/dL)**	0.03	0.73	**0.30**	**<0.01**	-0.09	0.31	0.02	0.81	0.08	0.30	0.04	0.56

BMI: Body mass index; WHR: Waist to hip ratio; HDL-C: High density lipoprotein; LDL: Low density lipoprotein; CRP: C-reactive protein; TG/HDL: Triglycerides/HDL-C index; Bold numbers indicate significant difference ($p<0.05$). *p* value by *Spearman* test.

Supplementary Table S4. Anthropometric, biochemical, and clinical variables in RA patients by calcidiol classification

Variables	Deficiency (<20ng/mL) (n=48)	Insufficiency (≥20ng/mL) (n=38)	Sufficiency (≥30ng/mL) (n=43)	p value
Age (years)[a]	48 (31-66)	47.5 (28-65)	48 (26-67)	0.8
Height(cm)[b]	1.6 ± 0.5	1.6 ± 0.6	1.6 ± 0.04	0.5
Weight (kg)[a]	67.6 (48.9-102.2)	65.2 (48.2-93.4)	66.2 (51-86.4)	0.9
BMI (kg/m^2)[a]	27.2 (20.1-38.5)	27.3 (18.7-36.6)	27.7 (19.7-35.5)	0.8
Waist circumference (cm)[a]	87.8 (68-108)	84.5 (69.8-116.4)	90.2 (67.5-108)	0.8
Hip circumference (cm)[a]	104.5 (91-127.5)	101 (86-123)	105.7 (86-132)	0.7
WHR (score) [a]	0.85 (0.74-0.95)	0.83 (0.76-1.01)	0.84 (0.73-0.98)	0.9
Glucose (mg/dL)[a]	92.7 (76.3-131.9)	87.3 (79-130)	87 (72.6-120)	0.2
Albumine (g/dL)[a]	3.91 (3.25-4.4)	3.98 (3.2-4.6)	3.94 (3.3-4.5)	0.2
Triglycerides (mg/dL)[a]	96.8 (50.9-188)	90 (56.9-159.9)	98.7 (32.6-213.2)	0.9
Cholesterol (mg/dL)[a]	175.3 (139.03-244.2)	164 (100.7-216)	171.5 (119.2-227)	0.1
HDL-C (mg/dL)[a]	47.6 (32.3-76.1)	49.1 (25.5-77.5)	49.9 (28.9-64)	0.8
LDL-C (mg/dL)[a]	98.3 (71.3-164)	92.9 (42-147.1)	94.5 (53.4-136.9)	0.1
Disease duration (years)[a]	6.7 (1-22)	6.5 (0.5-25)	5.5 (0.8-17)	0.7
DAS28 (ESR)[b]	3.7 ± 1.5	3.2 ± 1.3	3.9 ± 1.4	0.2
DAS28 (CRP) [a]	5.2 (0.7-34.3)	4.6 (0.2-40.9)	4 (0.3-25.7)	0.9
Remission (DAS28-CRP ≤2.6) [c]	39 (17/44)	27 (12/44)	34 (15/44)	0.8
Activity (DAS28-CRP > 2.6)[c]	36 (27/76)	31 (24/76)	33 (25/76)	
Tender joints [a]	1 (0-10)	2 (0-7)	1 (0-12)	0.8
Swollen joints [a]	0.5 (0-10)	1 (0-5)	0 (0-10)	0.8
CRP (mg/L) [a]	5.2 (0.7-34.3)	4.6 (0.2-40.9)	4 (0.3-0.5)	0.5
ESR (mm/h) [a]	36 (7-63)	43 (5-93)	36 (11-102)	0.8
ACPAs (UI/mL)[a]	122.5 (1.5-549)	228 (67-493.5)	100 (1-379)	0.6
RF (UI/mL) [a]	85 (16.6-160)	44.1 (9.6-177)	155.4 (11-2560)	**0.04**
Kannel index (LDL-C/HDL-C)[a]	2.2 (1.1-3.5)	1.7 (1-3.3)	2 (1-3.8)	0.1
Triglycerides/HDL-C ratio (score)[a]	2 (0.9-4.5)	1.9 (1-5.4)	2 (0.8-4.8)	0.7
Castelli atherogenic index (TC/HDL-C)[a]	3.7 (2.3-5.4)	3.2 (2.1-4.7)	3.4 (2.5-5.8)	0.1
sVDR (pg/mL)[a]	12.3 (0.7-104.6)	7.2 (0.02-139.4)	10.6 (0.02-132)	0.4
Calcidiol (ng/mL)[a]	14.9 (8.9-18.9)	24.7 (20.7-29.7)	36.3 (30.4-63.4)	**<0.001**
Calcitriol (pg/dL)[a]	48.1 (29.9-55.5)	43.5 (25.8-72.7)	41.3 (25.8-72.7)	**0.01**
Calcitriol/calcidiol ratio (pg/ng)[a]	3.1 (1.7-7.7)	1.7 (0.9-3.2)	1.1 (0.5-1.7)	**<0.001**

[a]Data provided in median (percentile: p5th–p95th), p value: Krukal Wallis test. [b]Data provided in media ± SD, p value: one-way analysis of variance (ANOVA). [c]Data provided in % (n), p value: F-fisher. BMI: Body mass index; WHR: Waist to hip ratio; HDL-C: High density lipoprotein; LDL-C: Low density lipoprotein; DAS: Disease activity score; CRP: C-reactive protein; ESR: erythrocyte sedimentation rate; ACPAs: Anti-citrullinated peptide antibodies; RF: rheumatoid factor; sVDR: soluble vitamin D receptor.
